# Development of a Vaccine against *Escherichia coli* Urinary Tract Infections

**DOI:** 10.3390/pathogens5010001

**Published:** 2015-12-31

**Authors:** Harry L. T. Mobley, Christopher J. Alteri

**Affiliations:** Department of Microbiology and Immunology, University of Michigan Medical School, Ann Arbor, MI 48109, USA; alteri@umich.edu

**Keywords:** *E. coli*, urinary tract infection, vaccine, antibody response

## Abstract

Urinary tract infection (UTI) is the second most common infection in humans after those involving the respiratory tract. This results not only in huge annual economic costs, but in decreased workforce productivity and high patient morbidity. Most infections are caused by uropathogenic *Escherichia coli* (UPEC). Antibiotic treatment is generally effective for eradication of the infecting strain; however, documentation of increasing antibiotic resistance, allergic reaction to certain pharmaceuticals, alteration of normal gut flora, and failure to prevent recurrent infections represent significant barriers to treatment. As a result, approaches to prevent UTI such as vaccination represent a gap that must be addressed. Our laboratory has made progress toward development of a preventive vaccine against UPEC. The long-term research goal is to prevent UTIs in women with recurrent UTIs. Our objective has been to identify the optimal combination of protective antigens for inclusion in an effective UTI vaccine, optimal adjuvant, optimal dose, and optimal route of delivery. We hypothesized that a multi-subunit vaccine elicits antibody that protects against experimental challenge with UPEC strains. We have systematically identified four antigens that can individually protect experimentally infected mice from colonization of the bladder and/or kidneys by UPEC when administered intranasally with cholera toxin (CT) as an adjuvant. To advance the vaccine for utility in humans, we will group the individual antigens, all associated with iron acquisition (IreA, Hma, IutA, FyuA), into an effective combination to establish a multi-subunit vaccine. We demonstrated for all four vaccine antigens that antigen-specific serum IgG represents a strong correlate of protection in vaccinated mice. High antibody titers correlate with low colony forming units (CFUs) of UPEC following transurethral challenge of vaccinated mice. However, the contribution of cell-mediated immunity cannot be ruled out and must be investigated experimentally. We have demonstrated that antibodies bind to the surface of UPEC expressing the antigens. Sera from women with and without histories of UTI have been tested for antibody levels to vaccine antigens. Our results validate iron acquisition as a target for vaccination against UTI.

## 1. Introduction

Urinary tract infection (UTI) is the second most common infection in humans after those involving the respiratory tract [1]. Half of all women will experience a symptomatic UTI, with incidences peaking in their early 20s. One-fourth of these women will experience recurrence within 6–12 months [2,3]. In the U.S., where the annual societal cost of UTIs is likely underestimated at $3.5 billion [4], four million women have UTIs continuously [5]. These infections range in severity from asymptomatic bacteriuria and cystitis to acute pyelonephritis and urosepsis, the latter of which can be fatal. 

This high frequency of infection results not only in huge annual economic costs, but in decreased workforce productivity and high patient morbidity [6]. At least 80% of these infections are caused by uropathogenic *Escherichia coli* (UPEC), which reside alongside commensal strains in the intestinal tract and gain access to the bladder via colonization of the urethra and to the kidney via ascension of the ureters. 

The urinary tract is among the most common sites of bacterial infection, and *E. coli* is by far the most common species infecting this site [7]. Individuals at high risk for symptomatic UTI include neonates, preschool girls, sexually active women, and elderly women and men. In 2006 (the most recent date for which comprehensive data are available), there were 11 million physician visits, over 1.7 million emergency room visits, and 479,000 hospitalizations of both men and women in the U.S. for UTI, at an annual cost of $3.5 billion [4,8]. These estimates place UTIs first among kidney and urologic diseases in terms of total cost.

Antibiotic treatment, typically with trimethoprim/sulfamethoxazole (TMP–SMX (*Bactrim*)) or ciprofloxacin, is generally effective for eradication of the infecting strain. However, documentation of increasing antibiotic resistance, allergic reaction to certain pharmaceuticals, alteration of normal gut flora, and failure to prevent recurrent infections represent significant barriers to treatment [9]. Indeed, treatment has been complicated by a rise in both the number of antibiotic-resistant strains and the prevalence of antibiotic-resistance mechanisms. For example, in the U.S. and Canada, 10%–25% of uncomplicated UTI isolates are resistant to TMP–SMX [9,10,11]. Even more troubling is the rate of multidrug resistance among UPEC isolates that has risen as physicians adapt prescription choices to address shifting microbial susceptibilities [12].

As a result, UTI prevention by vaccination represents a gap that must be addressed. Working toward a more effective and less costly alternative to antibiotic therapy for UTI management, we have identified protective antigens to specifically target UPEC. Although many women do experience recurrent UTI and UPEC heterogeneity complicates vaccine design, data from our animal model and human studies offer encouragement for successful UPEC vaccine development [13,14]. Immunization with four UPEC antigens stimulates increases in urinary and serum antibody titers that correlate with reductions in bladder and/or kidney bacterial load [15,16,17,18,19]. Based on preliminary studies, we are optimistic that an effective UPEC vaccine can be developed to combat or prevent human infections.

## 2. Existing UTI Vaccines

Conventional vaccinology approaches targeting virulence factors, such as FimH of type 1 fimbriae, have generated promising data in animal models [20], yet no vaccine is currently available in the U.S. for women with recurrent UTIs. A few products are marketed in other countries. Uro-Vaxom (OM Pharma, based in Switzerland) is currently marketed in Europe, Canada, and other countries [21]. It is indicated for prevention of acute UTI. The vaccine consists of extracts of 18 uropathogenic strains, packaged into a once daily oral tablet. A full course of vaccine requires three months to complete. Only modest protection is afforded by this product [22]. Solco-Urovac is a polymicrobial mixture of whole-cell, heat-killed uropathogens. *E. coli* comprises six of the 10 strains in the suppository vaccine. This vaccine, developed as a product similar to Uro-Vaxom, is purportedly being tested in humans at a university as of four years ago. However, no reports of this trial have emerged. Urvakol/Urostim is the product of a Czech and Bulgarian collaboration (BB-NCIPD Limited). They are seeking approval for a freeze-dried formulation of attenuated uropathogens in an oral tablet. The vaccine contains representative strains of *E. coli*, *Proteus mirabilis*, *Enterococcus faecalis* and *Klebsiella pneumoniae*.

*What is the cost of not having a UTI vaccine?* We have estimated the cost associated with recurrent UTIs based on data obtained over a three-year period at the University of Michigan Health System in different clinical settings. Data were extrapolated to the U.S. population (Table 1). These findings project the estimated total cost for recurrent UTI, in the U.S. alone, to be in excess of $5 billion.

*Who is the target population for this vaccine?* The primary target for the UTI vaccine would be women with recurrent UTIs. Approximately 11.3 million women get UTIs each year in the U.S., and *E. coli* accounts for 90% of these infections. The first line of therapy for uncomplicated UTI is treatment with the antibiotic TMP-SMX or nitrofurantoin for three days. Symptoms generally disappear after 48 h in 80% of cases. The remaining 20% will get another UTI, 30% of these recurrences will have yet another and 80% of the latter will have constant infections. Based on these data, we have identified the population of patients who can be targeted for the UTI vaccine to be the 1.1 million patients who get three or more incidences of UTI per year.

**Table 1:** Total recurrent UTI patient visits = 1.1 million.

Clinical Setting	Outpatient (88%)	Inpatient (6.5%)	Emergency Room (5%)
Number of Visits	970,000	72,000	55,000
Cost per Visit	$241	$64,824	$2843
Total Cost	$233 million	$4634 million	$156 million
Total Cost due to Recurrent UTI = $5.06 billion			

## 3. Progress towards Development of a Vaccine to Prevent UTI

To identify bacterial proteins for use as vaccine targets against UPEC infection, we employed a functional vaccinology approach, combining genomic, transcriptomic, and proteomic techniques. To begin, criteria defining potential UPEC vaccine targets were established and the screens described below were used to identify proteins meeting these parameters. Of the 5379 predicted proteins in prototype UPEC strain CFT073, only six proteins met all of our established criteria: (1) surface exposure (predicted and demonstrated), (2) induction during growth in human urine, (3) high *in vivo* expression in experimentally infected mice and (4) high *in vivo* expression in women with UTI, (5) immunogenicity, and (6) pathogen-specificity. Answering the following seven questions led us to selection of the vaccine candidate proteins.

### 3.1. What Secreted and Outer Membrane Proteins Can Be Predicted from the Genomic Sequence of E. coli CFT073?

Using the *E. coli* CFT073 genome sequence, we predicted proteins that potentially localize to the bacterial cell surface by identifying signal sequences, lipoprotein motifs, β-barrel structures, Hidden Markov surface prediction, integrin RGD (Arg-Gly-Asp), choline YY, transmembrane domains, and known outer membrane protein (OMP) motifs. Of 5379 predicted proteins, 343 proteins were identified as potentially surface-localized [15].

### 3.2. What Proteins Are Synthesized by E. coli CFT073 in Urine?

We used two-dimensional gel electrophoresis (2D-PAGE) and tandem mass spectrometry to characterize the uropathogenic *E. coli* (UPEC) outer membrane subproteome; 30 individual OMPs present on the bacterial surface during growth in human urine were identified. Fluorescence difference gel electrophoresis was employed to identify quantitative changes in levels of these UPEC strain CFT073 OMPs during growth in urine (<7 nM iron) [23]. Seven known receptors for iron compounds were induced in this environment: ChuA, IutA, FhuA, IroN, IreA, Iha and Hma [24]. Induction in human urine was verified using qRT-PCR. 

### 3.3. What Genes Are Expressed by E. coli CFT073 during Experimental Infection?

A CFT073-specific DNA microarray that includes each open reading frame (ORF) was used to analyze the transcriptome of CFT073 bacteria isolated directly from urine of experimentally infected CBA/J mice [25]. *In vivo* expression profiles were compared to *E. coli* CFT073 cultured statically to exponential phase in rich medium, revealing strategies this pathogen uses *in vivo* for colonization, growth, and survival in the urinary tract. The most highly expressed genes *in vivo* encoded translational machinery, indicating a rapid growth state despite specific nutrient limitations. Five iron acquisition systems, capsular polysaccharide and lipopolysaccharide synthesis, drug resistance, and microcin secretion were all highly upregulated during UTI. This study [25] represented the first assessment of any *E. coli* pathotype’s transcriptome *in vivo*.

### 3.4. Are the Same Genes Expressed by E. coli Infecting Women with UTI?

**Figure 1:**
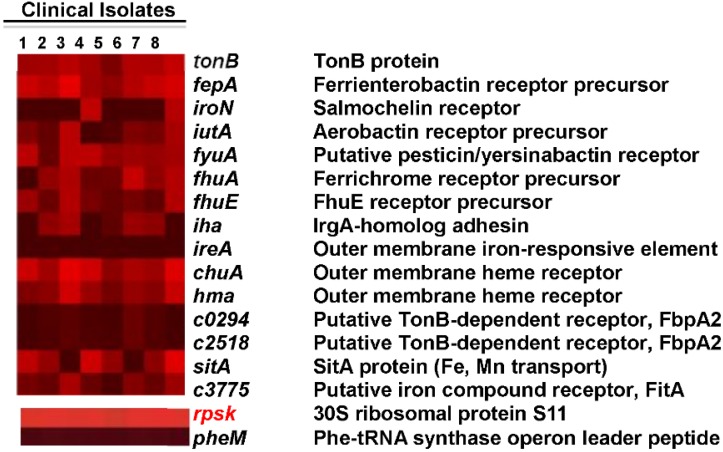
High levels of *in vivo* expression of UPEC iron acquisition genes. *E. coli* isolates were collected from the urine of eight women with active UTI. Heat map indicates normalized microarray signal intensities for genes encoding UPEC iron acquisition systems. For reference, the overall most (***rpsK*, +**) and least (***pheM*, −**) expressed genes are shown in the bottom panels, representing average signal intensities of 15.82 and 3.88, respectively [26].

Virulence gene expression was measured for *E. coli* in the urine of patients with UTI. We used the CFT073-specific microarray to measure global gene expression in eight *E. coli* isolates stabilized immediately after collection of urine from eight women presenting at the clinic with cystitis [26]. Gene expression profiles were compared to those of the same *E. coli* isolates cultured statically to exponential phase in pooled, sterilized human urine *ex vivo*. Known fitness factors, including iron acquisition, were highly expressed during human UTI (Figure 1). While these findings were highly correlated with data obtained from the murine *in vivo* transcriptome (*r* = 0.589; *p* < 0.0001) (described above), host-specific differences were observed. Findings presented in this study provide insight into the metabolic and pathogenic profile of UPEC in urine from women with UTI and represent the first transcriptome analysis for any pathogenic *E. coli* during a naturally occurring infection in humans [26]. RNA-Seq of RNA from *E. coli* collected (and stabilized immediately) directly from the urine of five women with documented uncomplicated UTI with symptoms of cystitis [27] verify that iron acquisition genes are expressed at high levels by *E. coli* during UTIs in women. While *ireA* was not present or well expressed in the women from the urology clinic cystitis patients, it was present and well expressed in RNA-seq samples from the women with uncomplicated cystitis and is prevalent in the collection of 315 *E. coli* strains [28].

### 3.5. What E. coli CFT073 Proteins Are Recognized by an Antibody Response during Experimental UTI?

We applied an immunoproteomics approach to vaccine development that has been used successfully to identify vaccine targets in other pathogenic bacteria [29]. Outer membranes were isolated from *E. coli* CFT073 cultured under conditions that mimic the urinary tract environment, including iron-limitation, osmotic stress, human urine, and exposure to uroepithelial cells. Outer membrane proteins were separated by 2D-PAGE and probed using pooled antisera from 20 CBA/J mice chronically infected with *E. coli* CFT073 [29]. Twenty-three outer membrane antigens reacted with the antisera and were identified by mass spectrometry. These antigens also included proteins with known roles in UPEC pathogenesis, such as ChuA, IroN, IreA, Iha, IutA, Hma, and FliC. Thus, an antibody response is directed against these virulence-associated factors during UTI. Also, genes encoding ChuA, IroN, Hma, and IutA are significantly more prevalent (*p* < 0.01) among UPEC strains as compared to fecal-commensal *E. coli* isolates [28]. Thus, conserved outer membrane antigens identified in this study could be rational candidates for a UTI vaccine designed to elicit protective immunity against UPEC infection.

### 3.6. What Surface-Exposed Motifs of E. coli CFT073 Proteins Are Susceptible to Limited Proteolysis?

To identify UPEC surface proteome during growth in human urine, whole-cell bacteria were labeled with a biotin tag to indicate surface-exposed peptides, subjected to limited proteolysis, and two-dimensional liquid chromatography-tandem mass spectrometry (2-DLC-MS/MS) [30]. This method discovered 25 predicted outer membrane proteins expressed by UPEC while growing in human urine. Nine of the 25 predicted outer membrane proteins were part of iron transport systems or putative iron-regulated virulence proteins as discovered above.

### 3.7. What Genes Are Common among UPEC Strains?

Based on the genome sequence of CFT073, microarrays were used for comparative genomic hybridization (CGH) analysis of a panel of uropathogenic and fecal/commensal *E. coli* isolates [31]. Genomic DNA from seven UPEC (three pyelonephritis and four cystitis) isolates and three fecal/commensal strains, including K-12 strain MG1655, was hybridized to the CFT073 microarray. The CFT073 genome contains 5379 genes. CGH analysis revealed that 2820 (52.4%) of the genes were common to all 11 *E. coli* strains, yet only 173 UPEC-specific genes were found by CGH to be present in all UPEC strains but in none of the fecal/commensal strains. When the sequences of three additional sequenced UPEC strains (UTI89, 536, and F11) and a commensal strain (HS) were added to the analysis, 131 UPEC-specific genes were identified that were not found in fecal/commensal strains [32].

## 4. Rational Selection of Vaccine Candidates

Of 5379 total genes in the CFT073 genome, 2368 genes were expressed *in vivo* (transcriptome), 343 genes were predicted to encode surface-exposed proteins, 131 genes were UPEC-specific, 23 genes encoded immunogenic outer membrane proteins, 10 genes were induced in urine, and eight genes encoded surface-exposed domains susceptible to limited proteolysis. Including the yersiniabactin receptor, FyuA, characterized from UPEC strain 536 [33], seven genes encoded proteins that met our criteria and comprised the final list of vaccine candidates.

### Testing of Top-Scoring Antigens, all Representing Outer Membrane Protein Components of Iron Acquisition Systems, as an Intranasal Vaccine

**Figure 2:**
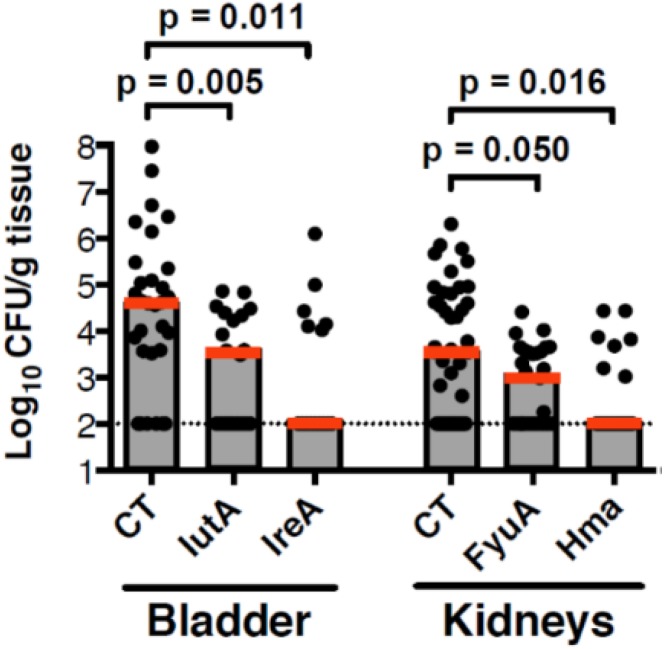
CBA/J mice were intranasally vaccinated with a primary dose of 100 µg purified protein crosslinked to 10 µg CT followed by two booster vaccinations with 25 µg protein. One week following the final boost, animals were transurethrally challenged with 1 × 10^8^ CFU of *E. coli* CFT073 or 536 and colonization was measured 48 h after challenge. Red bar is median value.

The seven vaccine candidates, ChuA, Hma, Iha, IreA, IroN, FyuA, and IutA, all belong to a functional class of molecules that is involved in iron acquisition, a process critical for pathogenesis in all microbes. Intranasal immunization of CBA/J mice with these outer membrane iron receptors elicited a protective systemic and mucosal immune response that significantly reduced colonization of the bladder and/or kidneys [15]. The cellular response to vaccination was characterized by the induction and secretion of IFN-γ and IL-17 [14]. Of the seven potential vaccine candidates, IutA, IreA, FyuA, and Hma provided significant protection from experimental infection (*p* < 0.035) (Figure 2). 

**Figure 3:**
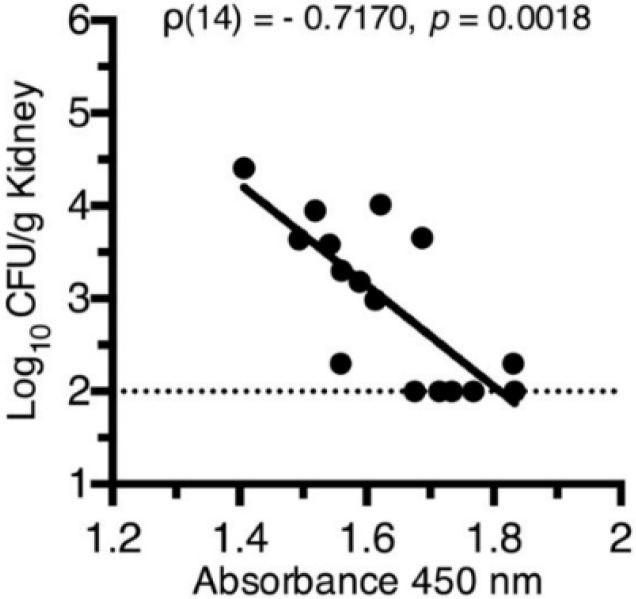
Correlation between vaccine-specific serum IgG titers and reduced bacterial counts in mice immunized with FyuA. Kidney CFUs from immunized and *E. coli*-challenged mice are plotted against their respective vaccine-specific serum IgG levels as measured by indirect ELISA, where absorbance at 450 nm reflects the relative quantity of vaccine-specific serum IgG. Dotted line indicates the limit of detection (100 CFU/g kidney tissue) for the immunization assay.

In immunized animals, class-switching from IgM to IgG (*p* = 0.0014) and production of antigen-specific IgA in the urine (*p* = 0.0165) both represent immunological correlates of protection from *E. coli* bladder colonization [15] (FyuA as an example, see Figure 3). While non-protective antigens elicited antibody production, these levels did not correlate with protection [15]. These findings are an important first step toward the development of a subunit vaccine to prevent UTIs and demonstrate how targeting an entire class of molecules that are collectively required for pathogenesis and induced *in vivo* may represent a fundamental strategy to combat infections.

## 5. Summary

We systematically identified four antigens that individually can protect experimentally infected mice from colonization of the bladder and/or kidneys by UPEC when administered intranasally with cholera toxin (CT) as an adjuvant [15]. To advance the vaccine for utility in humans, we will group the individual antigens (IreA, Hma, IutA, FyuA) into an effective combination to establish a multi-subunit vaccine. We will also optimize the most effective adjuvants and routes of inoculation currently used for immunization of humans. 

In our approach, we need to formulate the most efficacious combination of the four antigens identified in our ongoing studies (IreA, Hma, IutA, and FyuA) using the most effective adjuvants, doses, and routes that have been used successfully in human vaccines. When completed, it will be important to identify the mechanism of protection provided by the optimal antigen combination, adjuvant, dose, and route of administration to move forward to clinical trials. We expect to identify the optimal combination of pathogen-specific antigens that will protect against development of UTI caused by UPEC and to understand the mechanism by which protection is afforded to the host. The development of a vaccine to prevent this public health scourge in women with recurrent UTI and those susceptible to their first UTI would improve the quality of life for millions of women.
